# Congenital Diarrhoeas and Enteropathies

**DOI:** 10.3390/nu16172971

**Published:** 2024-09-03

**Authors:** Jutta Köglmeier, Keith James Lindley

**Affiliations:** Department of Paediatric Gastroenterology, Great Ormond Street Hospital for Children NHS Foundation Trust, Great Ormond Street, London WC1N 3JH, UK; keith.lindley@gosh.nhs.uk

**Keywords:** diarrhoea, enteropathy, congenital, children, diagnosis, classification

## Abstract

Congenital diarrhoeas and enteropathies (CODE) are a heterogeneous group of disorders. Many affected infants present with catastrophic dehydration in the first few days of life, although the clinical phenotype is variable. Advances in the understanding of underlying pathomechanisms and genetic testing, as well as improved management, in particular intravenous nutrition support, have allowed affected patients to survive well beyond childhood. Awareness and understanding of these rare diseases are hence needed, both amongst paediatricians and adult physicians. In this review, we discuss the different groups of disorders based on a review of the current literature and provide a diagnostic and therapeutic approach. Many of the subtypes of CODE result in the need for prolonged or indefinite parenteral nutrition. Further research is needed to identify new CODE to improve the recognition and management of these children, which can assist in developing new targeted therapies and potentially a long-term cure.

## 1. Introduction

It is over 50 years since Avery’s first description of a syndrome of intractable diarrhoea in infancy [1]. During this period, great progress has been made in our understanding of the genetics and cell biology of this heterogeneous group of disorders, and both supportive and interventional treatments have advanced significantly. Improvements in therapies and nutritional support are such that many of these children are now surviving into adulthood, providing new and challenging horizons for our adult gastroenterology colleagues.

**Figure 1:**
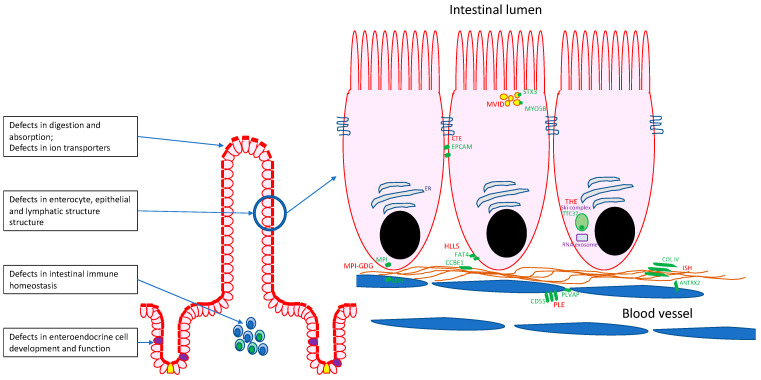
Classification of CODE according to underlying defect. Genetic defects are in green, and clinical phenotypes are in red. CTE—congenital tufting enteropathy; MVID—microvillus inclusion disease; THE—tricho-hepato-enteric syndrome; MPI-GDG—congenital disorder of glycosylation type 1b; PLE—syndromic protein-losing enteropathies; ISH—infantile systemic hyalinosis; HLLS—Hennekam lymphangiectasia lymphoedema syndrome.

Congenital diarrhoeal disorders are genetically and phenotypically heterogeneous. In recent years, a useful classification of these disorders has been suggested, which provides a helpful framework within which to discuss this heterogeneous group [2]. These authors propose four groups of disorders: (i) defects in digestion and absorption of nutrients or of ionic transport, (ii) defects in enterocyte structure and function, (iii) defects in enteroendocrine cell differentiation and (iv) defects in intestinal immune homeostasis (see Figure 1).

Many of these disorders present with catastrophic dehydration in the first few days of life, although the clinical spectrum of disease is diverse. After resuscitation and ongoing management of electrolyte/pH disturbances/losses, it is generally good clinical practice to keep the infants nil by mouth on intravenous fluids for 24 h in the first instance. This will allow the clinician to distinguish between osmotic (malabsorptive) and secretory diarrhoeal phenotypes, as osmotic diarrhoea will stop when the infant is fasting.

Osmotic diarrhoea is caused by the malabsorption of solutes, and the stool osmotic gap is high. Secretory diarrhoea is the consequence of the active secretion of ions into the lumen of the intestine, and stool osmotic gap is usually low. Stool osmotic gap is calculated with the following formula: 290 − 2 × (stool sodium + potassium) and considered low if less than 50 mOsm and high if above 100 mOs [3]. Furthermore, the measurement of stool sodium (Na^+^) concentration (in a well-hydrated infant) will confirm if the diarrhoea is secretory if the stool Na^+^ is above 80 mml/L. Where it is difficult to distinguish stool from urine, then insertion of a nasogastric tube into the rectum and gentle suction on a syringe will commonly yield Na^+^-rich fluids in secretory diarrhoeas. Attention to the history is also important at this stage. A history of maternal polyhydramnios, premature delivery and failure to pass meconium suggests an intrauterine secretory process and a likely primary ion transporter defect. The passage of meconium with the onset of diarrhoea after a few days or even weeks is suggestive of an epithelial disorder, the phenotype of which is highly variable. Later onset is usually inflammatory. Where the infant has passed meconium and the diarrhoea stops on fasting, an osmotic diarrhoea is more likely, either due to a primary defect in the digestive/absorptive apparatus or occasionally an enteropathy. In these infants, the measurement of stool osmolality and sodium after a feed should illustrate the presence of an osmotic gap with low stool sodium, and stool sugar chromatography should confirm the nature of the osmotically active foodstuff/substance. Other presentations include mixed pictures, protein-losing enteropathies, bloody diarrhoea and selective fat malabsorption. A detailed family history noting the presence/absence of consanguinity is important, as is attention to other phenotypic features, including dysmorphism, hair abnormalities, skin rashes and perineal pathologies. Some children will have an onset outside of the neonatal period.

Most children will require a small intestinal biopsy as part of the diagnostic evaluation, allowing the assessment of the epithelial structure and cell types as well as villus height and depth of crypts. In addition to obtaining biopsies for creating paraffin sections, it is good practice to take additional small bowel biopsies for electron microscopy and snap freezing for subsequent immunohistochemistry or other stains, as indicated. The morphological features characteristic of some congenital diarrhoeas are not always prominent in the first month of life, and on occasion, a follow-up biopsy when the child is older is necessary for a histological diagnosis. In many institutions, including our own, DNA can be sent to screen a panel of genes associated with early-onset diarrhoea. Increasingly, whole-exome or whole-genome sequencing is being undertaken where the diagnosis is unclear on initial investigation [4,5].

For the purposes of this short review, defects in intralumenal digestion are not discussed, but the reader is reminded that exocrine pancreatic insufficiency is amongst the commonest causes of malabsorption in infancy. Neither is this review exhaustive, and the reader is referred to other more encyclopaedic texts [2,3,6,7,8].

## 2. Materials and Methods

A literature search was performed from 1992 to 2022 using Pubmed, MEDLINE and Cochrane Database of Systematic Reviews using the search terms ‘diarrhea, enteropathy, congenital, children, diagnosis and classification’. A total of 52 papers were considered relevant and referenced in this review to give an overview of the current evidence available.

### 2.1. Defects in Epithelial Ionic Transport or Digestion and Absorption of Nutrients

(a) Congenital chloride-losing diarrhoea (CCD)

This is the result of biallelic mutations in the SLC26A3 gene (alias DRA) on chromosome 7q31, which encodes the enterocyte apical membrane HCO_3_^−^/Cl^−^ exchanger (reviewed in [8]). The disease is seen most commonly in Finnish, Polish and Arabic communities but occurs sporadically elsewhere. Infants present with intrauterine diarrhoea with visibly distended bowel loops on antenatal ultrasound and polyhydramnios, which frequently results in premature delivery. Characteristically, the infants fail to pass meconium and rapidly dehydrate due to stool losses of typically 200–300 mL/kg/24 h. The characteristic biochemical disturbance that arises through the loss of acidic chloride-rich stools and secondary hyperaldosteronism is a blood hypochloraemic hypokalaemic alkalosis with rapid progression to pre-renal failure. The measurement of stool chloride (>90 mmol/L) is usually diagnostic, although, in severely dehydrated infants, the resuscitation and correction of blood biochemical abnormalities may be necessary for the classic stool electrolyte abnormalities to manifest.

Treatment revolves around the correction of hydration and electrolyte/acid-base disturbances with intravenous fluids and then a transition to oral electrolyte supplementation. Intravenous resuscitation of the entire fluid deficit should be with normal saline, and then ongoing stool losses should be replaced with normal saline. Maintenance fluids (5% dextrose) will require (in addition to the addition of maintenance Na^+^ and K^+^) added potassium to make up the entire calculated K^+^ deficit in the first 24 h. On transition to oral salt supplementation, children aged <3 years generally require 6–8 mmol/kg/day of chloride (molar ratios of Na^+^:K^+^ 7:3), and children aged >3 years require 3–4 mmol/kg/day (molar ratios of Na^+^:K^+^ 1:1). It has been suggested that dietary supplementation with butyrate might increase colonic salvage of Cl^−^ in these children, but clinical results have generally been disappointing. Suggestions that proton pump inhibitors might reduce stool losses of electrolytes have also proven clinically disappointing. It is important to check the adequacy of salt supplementation by measuring urinary Na^+^ and Cl^−^ at routine clinic visits [9].

A major danger to these children is rapid dehydration with intercurrent episodes of gastroenteritis. Failure to appreciate the need for early intravenous salt replacement in such instances can lead to recurrent acute kidney injury. This and chronic hyperaldosteronism as a result of inadequate salt replacement result in a high incidence of chronic renal disease in these children.

(b) Congenital sodium diarrhoea (CSD)

(i) “Classical” CSD presents antenatally, similar to CCD, but is much rarer and is genetically heterogeneous, with syndromic and non-syndromic forms [9]. CSD arises as a result of inadequate functioning of the apical enterocyte membrane sodium proton antiporter 3 (NHE3), a Na^+^/H^+^ exchanger. Biallelic mutations in the gene SLC9A3 (seen in 40% of infants with CSD) can result in the absence or dysfunction of NHE3, with diarrhoea of antenatal onset presenting as polyhydramnios, dilated loops of bowel and premature delivery. As with CCD, there is failure to pass meconium, rapid postnatal dehydration and, in this instance, hyponatraemic acidosis with high stool sodium.

NHE3 can be downregulated by elevated intracellular cyclic guanosine monophosphate (cGMP) concentrations, as seen with activating mutations of the GUCY2C gene, which encodes receptor guanylate cyclase C. Dominant activating mutations in the GUCY2C gene cause classical CSD in about 20% of cases of CSD. Approximately 40% of infants with classical CSD have a genetic cause that is presently undefined. Classical CSD is associated with early- and late-onset forms of inflammatory bowel disease [10].

(ii) “Syndromic” CSD comprises a group of infants with choanal atresia and recurrent punctate keratitis. Less commonly, bowel and anal atresias, polydactyly and cleft palate are seen. In contrast to “classical” CSD, small bowel biopsy demonstrates villus atrophy and epithelial tufting (“tufting enteropathy”). Diarrhoea arises as a result of the misregulation of the heterotrimeric epithelial sodium channel, which is critical for sodium reabsorption in the distal colon but is expressed throughout the gastrointestinal tract. It arises as a consequence of a mutation in the gene SPINT2, which encodes a serine peptidase inhibitor. The activity of ENaC depends upon its proteolytic activation by the matriptase–prostasin system. SPINT2 targets the serine proteases matriptase and prostasin. The loss of SPINT2 from the intestinal epithelium of the small and large intestines results in a decrease in the cell surface expression of matriptase and, as a result, a decrease in epithelial ENaC expression.
(c) Congenital Glucose–Galactose malabsorption (GGM)

This is an autosomal recessive disorder due to mutations in the SLC5A1 gene on chromosome 22q13.1 encoding SGLT1, the apical enterocyte membrane sodium-coupled glucose/galactose transporter [11,12]. Affected infants are apparently healthy at birth but, following a feed of either breast milk or a breast milk substitute, develop torrential watery diarrhoea and dehydrate rapidly. When feeding is stopped, the diarrhoea stops rapidly. Analysis of the diarrhoeal stool reveals the presence of reducing sugars and low stool sodium with an osmotic gap (due to malabsorbed glucose). Stool sugar chromatography demonstrates the stool to be full of glucose (a hydrolysis product of milk lactose). Oral rehydration solutions (ORSs) utilise SGLT1 to drive water absorption into the intestine, but the use of these in infants with GGM precipitates diarrhoea again. Small bowel biopsies in these infants are histologically normal. Treatment simply involves avoiding milks/foods containing glucose, galactose or polysaccharides, which are digested into these sugars within the gastrointestinal tract. Fructose is not absorbed through SGLT1 but by a different apical membrane transporter (GLUT5). Feeding these infants a fructose (rather than lactose)-based infant formula does not cause diarrhoea, and on this feed, the infants thrive. The sodium-coupled glucose transporter in the kidney is SGLT2, so glycosuria is not part of the clinical picture at presentation in these infants. There is, however, an association with renal tubular acidosis, which can give rise to nephrocalcinosis in later life.

Acute infectious diarrhoea can be challenging in these infants/children, as conventional ORSs will exacerbate water loss in the stools. Fructose (unlike glucose) absorption is not driven by the electrochemical gradient across the apical enterocyte membrane, and so an ORS based on fructose is unlikely to be effective in bringing about rapid rehydration of infants with infectious diarrhoea, and usually, these infants will need the expeditious use of intravenous rehydration.

(d) Congenital lactase deficiency

Congenital lactase deficiency (CLD) is an extremely rare autosomal recessive disorder (chromosome 2q21) mostly found in Finland. Lack of intestinal epithelial lactase phlorizin hydrolase (LPH) in the villous brush border results in an inability to digest lactose to glucose and galactose. LPH also has general hydrolase and β-glucosidase activities. Clinical presentation is very similar to GGM, with diet-induced watery diarrhoea starting from the first (lactose-containing) feed, which can result in rapid dehydration. Morphologically, the duodenum is normal, and the activities of other brush border disaccharidases are preserved. CLD is distinct from adult-type hypolactasia (ATH), which affects 95% of adults worldwide, with the exception of northern Europeans, who have a lactase-persistence phenotype [13]. In ATH, intestinal lactase activity usually declines from around the age of 5 years to typically <10% of infant activity.

(e) Sucrase-isomaltase deficiency

This is again rare, except in some indigenous populations of Greenland and Canada. The clinical phenotype is highly variable, with symptoms dating from the introduction of sucrose/glucose polymer into the diet. This is usually at weaning but can be earlier, when feed thickeners containing glucose polymers are given to infants with symptomatic gastroesophageal reflux. The reader is referred elsewhere for a fuller description [4].

(f) DGAT1 deficiency

Acyl CoA:diacylglycerol acyltransferase 1 (DGAT1) catalyses the final step in the re-esterification of triglycerides within the enterocyte before packaging into chylomicra and secretion into the lymphatics. Homozygous DGAT1 deficiency is associated with congenital diarrhoea and hypoproteinaemia secondary to protein-losing enteropathy [14]. The mechanism of both the diarrhoea and the protein loss is unknown, although enhanced lipid toxicity has been suggested. Treatment with a low-fat diet normalises faecal protein losses [15].

(g) Other defects in intestinal epithelial lipid re-esterification and export

This group of disorders includes abetalipoproteinaemia, hypobetalipoproteinaemia and chylomicron retention disease. Abetalipoproteinaemia and familial hypobetalipoproteinaemia are rare diseases characterised by hypocholesterolaemia and malabsorption of lipid-soluble vitamins, leading to retinal degeneration, neuropathy, and coagulopathy. Microsomal triglyceride transfer protein (MTP) is required for lipoprotein assembly within the enterocyte following the re-esterification of lipids and is defective in abetalipoproteinaemia [16]. Hypobetalipoproteinaemia is due to homozygous mutations in the APOB gene, resulting in improper packaging and secretion of apolipoprotein B-containing particles. Small bowel histology will show normal villous architecture with vacuoles in the apical villous cytoplasm that can be highlighted with fat stains such as Sudan Red.

Steatorrhoea is usual, although most patients with abetalipoproteinaemia present later in childhood, many of the symptoms being attributable to the effects of vitamin E deficiency. The few patients described with hypobetalipoproteinaemia have presented in adulthood.

Chylomicron retention disease (CRD) is an autosomal recessive disorder of severe fat malabsorption associated with failure to thrive in infancy [17]. Also known as Anderson disease, CRD is caused by homozygous or compound heterozygous mutations in the SAR1B gene encoding the so-called secretion associated Ras related GTPase 1B. This molecule is involved in the transport of immature chylomicrona from the endoplasmic reticulum to the Golgi apparatus within the enterocyte, and the mutation results in the accumulation of lipids within the enterocyte. Clinical presentation varies but usually involves diarrhoea, which can be severe, steatorrhoea and growth retardation. Histology of the small bowel again shows vacuolisation of villous enterocytes due to intracellular lipid accumulation, with normal enterocytes in the crypts.

**Figure 2:**
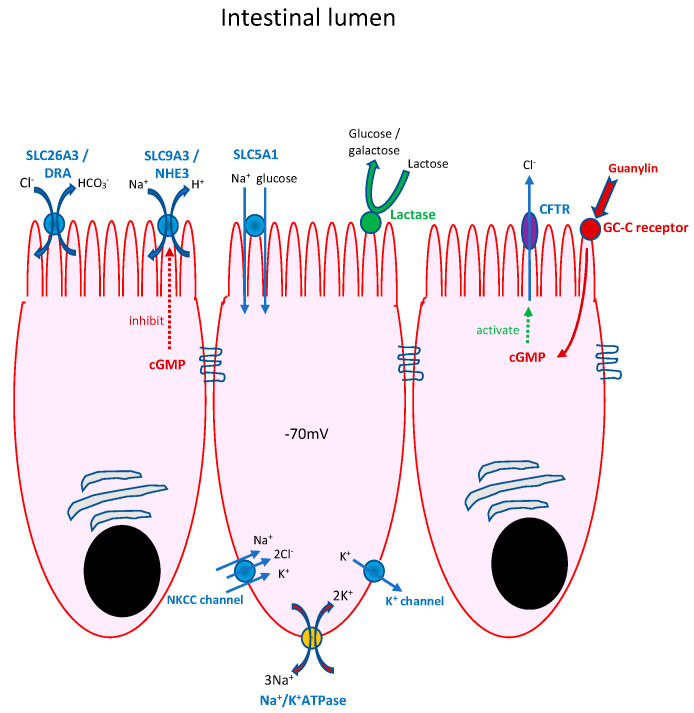
Schematic representation of small bowel enterocytes depicting some of the apical and basolateral ion channels/pumps/enzymes. SLC26A3—chloride/bicarbonate antiporter; SLC9A3—sodium/hydrogen antiporter; SLC5A1—sodium/glucose cotransporter; CFTR—cystic fibrosis transmembrane receptor (see text for further details).

A schematic representation of small bowel enterocytes depicting some of the apical and basolateral ion channels/pumps/enzymes discussed in the text above is shown in Figure 2.

### 2.2. Defects in Epithelial Structure

(a) Microvillus inclusion disease

Microvillus inclusion disease (MVID) causes a severe secretory diarrhoea that starts in the early postnatal period. Affected infants usually pass meconium but go on to develop a high-volume (typically 300 mL/kg/24 h) electrolyte-rich secretory diarrhoea resulting in rapid-onset severe dehydration. First described in 1978, this disease is characterised by a small bowel crypt hypoplastic villus atrophy with PAS-positive cytoplasmic inclusions on light microscopy, which on electron microscopy are evident as an intra-cytoplasmic brush border containing cystic structures (microvillus inclusions).

Most patients with early-onset MVID have inactivating mutations in the gene encoding myosin 5b (MYO5B, chromosome 18q21.1) [18]. There is wide variation in clinical phenotype, particularly with regard to the presence or absence of early-onset cholestasis. Interestingly, MYO5B mutations have been described in familial intrahepatic cholestasis in individuals without intestinal disease. Mutations in MYO5B result in the uncoupling of specific RAB small GTPases from their normal sub-apical membrane location, abnormal localisation of RAB8A and RAB11A throughout the cytoplasm and the formation of intracytoplasmic microvillus inclusions. MYO5B knockout mice have reduced expression of NHE3, SLC26A3, SGLT1 and AQP7 channels/exchangers at the apical membrane but preserved CFTR, implying reduced ability to absorb sodium, chloride and water but maintained ability to secrete chloride. These observations might explain why these children respond poorly to oral electrolyte supplementation (cf chloride-losing diarrhoea) and are dependent lifelong upon intravenous nutrition/hydration. PN is often challenging in these children because of profound electrolyte and water losses. Many will progress to bowel transplantation as definitive therapy when adequate supplementation with fluid and electrolytes is no longer possible or early complications such as recurrent life-threatening central venous catheter (CVC)-related bloodstream infections associated with CVC change and loss of central venous access sites, as well as IF-related liver disease, occur.

Whole-exome sequencing has demonstrated a few children with a milder phenotype to have mutations in syntaxin 3 (STX3), which is important in the maintenance of cell polarity in the intestine, and others to have mutations in syntaxin binding protein 2 (STXBP2).

(b) Tufting enteropathy

First described in the 1990s, this disorder starts variably within the first month of life with secretory diarrhoea. The clinical phenotype varies widely in different populations, although the majority of children will require parenteral nutrition to maintain normal nutritional status and growth. Tufting enteropathy (TE) is most common in Arabic and North African populations. Histologically, the disease is characterised by the presence of epithelial tufts anywhere between the duodenum and the rectum. The pathognomonic histopathological changes will sometimes become apparent earlier in the large bowel than in the small bowel. Histochemical stains for EpCAM are frequently negative (see below). Mucosal inflammation is a frequent finding in biopsies from these children.

There appear to be both syndromic and non-syndromic forms of the disease. Non-syndromic forms are associated with mutations in the epithelial cell adhesion molecule gene (EPCAM) found on chromosome 2q21 [19]. EpCAM is an important regulator of intestinal epithelial cell differentiation, proliferation and migration. The syndromic form of the disease is associated with mutations in the SPINT2 gene encoding a serine protease inhibitor, which is important in epithelial regeneration [20]. The spectrum of associated features includes superficial punctate keratitis and choanal atresia and, less commonly, bowel atresias and polydactyly. Changes in the activity of the epithelial sodium channel (ENaC) have been described in the congenital sodium diarrhoea section.

Histology seems to correlate poorly with outcome, with lifelong PN dependence not being inevitable [21].

(c) Trichohepatoenteric syndrome (Phenotypic or Syndromic Diarrhoea)

These patients have a triad of chronic diarrhoea, facial dysmorphism and hair abnormalities (woolly thickened hair prone to breakage—trichorexis nodosa). Many children are of small gestational age. The mechanism of the diarrhoea is ill-understood, although villous atrophy of the small intestine with or without an inflammatory infiltrate is not uncommon. Diarrhoea usually starts within the first 6 months of life with failure to thrive. Skin abnormalities, cardiac defects, liver disease and platelet anomalies are sometimes seen. Immune abnormalities are common and varied, including hypogammaglobulinaemia. Switched memory B-lymphocyte count is typically very low, IFN-γ production by T and NK cells is impaired and associated with a reduced degranulation of NK cells, and T-cell proliferation is frequently abnormal [22]. Non-specific liver dysfunction is common, and the prognosis is often poor, with death before the 5th birthday frequently associated with liver disease.

Gene mutations in TTC37 and SKIV2L have been found in affected individuals. TTC37 encodes a protein integral to the process of exosome-mediated RNA surveillance, as does SKIV2L. The link between mRNA surveillance pathways and protracted diarrhoea is currently poorly understood, and mutations can be present without clinical diarrhoea. Patients lacking SKIV2L seem to have more severe disease than those lacking TTC37 [23].

(d) Syndromic protein-losing enteropathies (PLEs) with early-onset diarrhoea

Excessive enteric protein loss occurs as a result of (i) protein leakage from abnormal lymphatics, (ii) abnormalities of extracellular matrix charge facilitating extravasation of proteins from the circulation and (iii) small bowel inflammation. It is important to definitively distinguish between enteric protein loss and protein maldigestion as a cause of hypoproteinaemia, especially in young infants when there is little to distinguish the very different causes symptomatically. Simple measurements of faecal α_1_-antitrypsin and stool elastase will usually suffice to make this important distinction. Congenital protein-losing enteropathies are genetically heterogeneous. Careful examination of the patient to look for associated phenotypic abnormalities is essential.

(i) Plasmalemma Vesicle-Associated Protein mutations

Plasmalemmal vesicle-associated protein (PVALP) is an endothelial cell-specific protein that is required for the synthesis of the stromal and fenestral diaphragms of blood vessels. The protein is involved in the regulation of blood vessel permeability. Nonsense mutations in the PVALP gene have been associated with a severe syndromic form of PLE [24]. The index infant had evidence of an in utero cystic hygroma and facial oedema. At birth, the infant was dysmorphic with hypoplastic supraorbital bulges, low-set ears, a flattened, elongated and prominent philtrum, anteverted nostrils, downturned corners of the mouth, a high-arched palate and moderate generalised oedema. He had multiple iris cysts on ophthalmologic examination and undescended testes. The infant developed intractable secretory diarrhoea on day 8 of life with hyponatraemic, hypomagnesaemic hypocalcaemic metabolic acidosis. Attenuated forms of the disease that present later in life are also recognised [25].

(ii) Hennekam lymphangiectasia–lymphoedema Syndrome

**Scheme 1:**
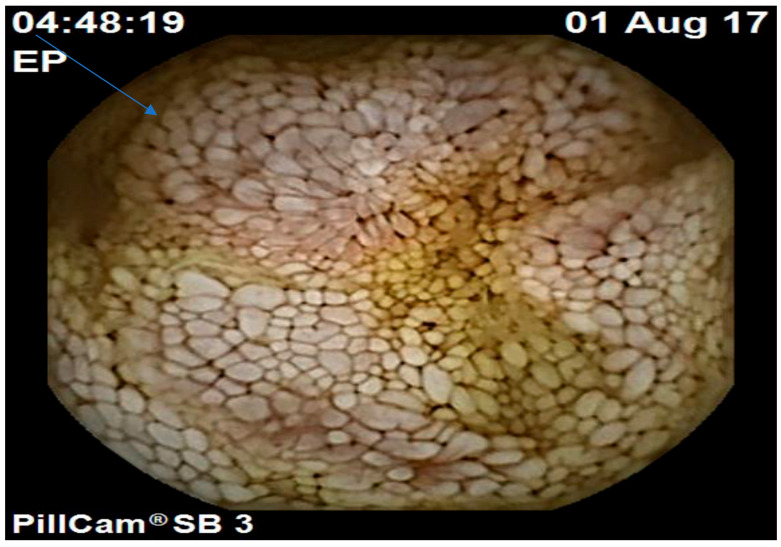
Capsule endoscopy image showing extensive intestinal lymphangiectasia (with permission).

**Scheme 2:**
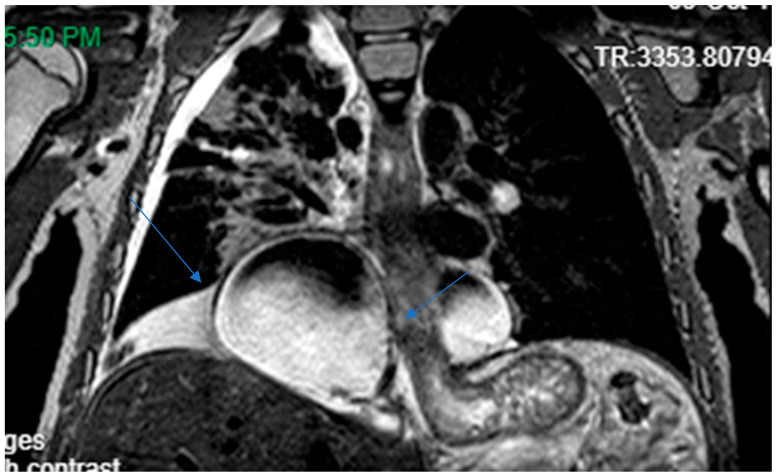
MRI chest showing right basal cystic lesion extending into medial left lung (with permission).

Hennekam lymphangiectasia–lymphoedema syndrome (HLLS) is a genetically heterogeneous autosomal recessive disorder characterised by generalised lymphatic dysplasia of the intestine, limbs, pericardium and pleural space (see Scheme 1 and Scheme 2). Affected children suffer congenital lymphoedema, facial dysmorphism and variable intellectual disability [26]. Facial anomalies included a flat face, flat nasal bridge, hypertelorism, epicanthal folds, small mouth, tooth anomalies, and ear defects (Scheme 2). Hypothyroidism is not infrequent.

A number of different genes that control endothelial vascular development have been associated with HLLS, including CCBE1 (collagen and calcium-binding epidermal growth factor domain-containing protein 1)—found in 25% of cases—FAT4, ADAMTS3 and FBXL7. The cadherin Fat4 controls endothelial cell polarisation during lymphatic valve morphogenesis [27]. FBXL7 signalling is also involved in the Fat4 pathway. CCBE1 acts via ADAMTS3 (a disintegrin and metalloprotease with thrombospondin motifs-3 protease) to enhance vascular endothelial growth factor C signalling.

The gastrointestinal phenotype is extremely variable to include both severe infantile presentations and presentation later in the first decade of life. Severe phenotypes can suffer intrauterine hydrops, ascites and be PN-dependent. Invariably, there is protein-losing enteropathy and lymphangiectasia with associated lymphopenia, and hypogammaglobulinaemia is usual. Small bowel biopsy will show features of lymphangiectasia but may also be inflamed [28].

(iii) Congenital disorder of glycosylation type 1b (MPI-CDG)

MPI-CDG is an autosomal recessive disorder of glycosylation due to mutations in the PM1 gene encoding phosphomannose isomerase (PMI). PMI is a cytosolic enzyme catalysing the isomerisation of fructose 6-phosphate to mannose 6-phosphate.

Gastrointestinal symptoms are prominent at presentation with vomiting, intractable diarrhoea, protein-losing enteropathy (PLE) and usually villus atrophy on small bowel biopsy. There is usually hepatomegaly with fibrosis resembling congenital hepatic fibrosis and hamartomatous collections of bile ducts on liver biopsy [29,30]. Subtle phenotypic features that are sometimes present on clinical examination include inverted nipples, abnormal subcutaneous fat distribution and rather prominent heels when the feet are examined.

Biochemically, there is usually hypoalbuminaemia with a mild transaminitis. Importantly, coagulation is usually deranged, as the glycosylation of factor XI, antithrombin III and proteins C and S is defective, and blood levels are low. These deficiencies will need to be addressed before a small bowel biopsy is undertaken. Thrombosis, as well as life-threatening bleeding, can occur. Mild hyperinsulinaemic hypoglycaemia is frequent.

Transferrin iso-electric focussing will reveal a characteristic abnormality in PMI deficiency, and the diagnosis can be confirmed by genetic sequencing of the PMI gene.

The disorder has been successfully treated with oral mannose supplementation, with improvements in both diarrhoea and liver function [31].

(iv) Hereditary PLE in association with CD55 deficiency (CHAPLE syndrome)

Deficiency of the complement inhibitor CD55 is associated with early-onset diarrhoea, severe protein-losing enteropathy with lymphangiectasia and susceptibility to large vein thrombosis. The disorder is clinically heterogenous but can present in the first year of life with oedema, hypoalbuminaemia malabsorption, malnutrition, recurrent infections and occasionally bowel inflammation [32]. This has been given the acronym CHAPLE syndrome (complement hyperactivation, angiopathic thrombosis and protein-losing enteropathy). It appears that CD55 has a role in adaptive immune regulation, and T cells from deficient patients overproduce TNF and underproduce IL10.

Eculizumab therapy (which suppresses C5a production) in a family with CHAPLE syndrome reduced enteric protein loss and bowel movement frequency [33]. Whilst eculizumab decreased the size of intestinal lymphatics within 2½ months of therapy, it is unclear to what extent the lymphangiectasia is primary or secondary.

(v) Infantile Systemic Hyalinosis

Infantile systemic hyalinosis (ISH) is a rare autosomal recessive disorder and an allelic form of hyaline fibromatosis syndrome (HFS) that is caused by mutations in the ANTRX2 gene (previously designated capillary morphogenesis gene 2) encoding the transmembrane anthrax toxin receptor 2. ANTRX2 is implicated in the regulation of angiogenesis and extracellular matrix turnover/uptake via matrix metalloproteinases. Mutations in ANTRX2 can result in the accumulation of extracellular collagen IV, which accumulates in ISH and HFS. 

Clinical features of ISH, largely attributable to extracellular hyaline deposits, include characteristic skin lesions with subcutaneous nodules, progressive joint flexion deformities, painful swollen joints, gingival overgrowth, persistent diarrhoea with protein-losing enteropathy, and failure to thrive due to the accumulation of hyaline material in multiple organs [34,35].

### 2.3. Enteroendocrine Cell Defects

Enteroendocrine cells (EECs) are present throughout the gastrointestinal tract and produce hormones that act locally or systemically. This group of disorders generally presents with osmotic diarrhoea with or without extraintestinal endocrine abnormalities.

(a) Enteric anendocrinosis

The development of pancreatic β-cells and intestinal EECs is regulated by a basic helix-loop-helix transcription factor, NEUROG3. Children with neurogenin 3 deficiency present with neonatal diabetes and osmotic diarrhoea [36]. Small bowel biopsies are morphologically normal, but staining for chromogranin 3 will show EECs to be absent.

(b) Mitchell–Riley Syndrome

Regulatory factor 6X (RF6X) is a transcription factor downstream of NEUROG3. Homozygous mutations in RFX6 can have duodenal atresia, biliary abnormalities, neonatal diabetes and malabsorptive diarrhoea. In contrast to enteric anendocrinosis, EECs are present within the gut, as RF6X is downstream of NEUROG3.

(c) ARX deficiency

Loss of function mutations in the ARX gene result in deficiency of the homeobox protein ARX and are associated with X-linked mental retardation, seizures, lissencephaly and abnormal genitalia with or without diarrhoea [37]. ARX is a downstream target of NEUROG3 and is expressed in EECs that express CCK, secretin and glucagon. The neurological and gastrointestinal manifestations of this disorder are highly variable, but most affected children die before the age of 6 years.

(d) Proprotein convertase (PPC) 1/3 deficiency—enteric dysendocrinosis

Pro-hormones produced by enteroendocrine cells are cleaved into active hormones by PPC1/3. Homozygous mutations in the PCSK1 gene (which encodes PPC1/3) cause malabsorptive diarrhoea and other endocrinopathies, including hypothyroidism, hypoadrenalism and hypogonadism. Proinsulin levels in the circulation are usually extremely high. Intestinal failure with PN dependency is not uncommon in infants with PPC1/3 deficiency. PPC1/3 is also expressed in the hypothalamus, and deficient individuals are usually hyperphagic, resulting in obesity in older children. Our personal experience with these children is that the calorie requirement for normal rates of weight gain is often diminished [38].

(e) ICR-related gastrointestinal endocrinopathy

A recent investigation of 8 children with congenital diarrhoea from 7 unrelated families using whole-genome sequencing has discovered a non-coding region on chromosome 16, termed the “intestine critical region” (ICR), which appears to contain gene regulatory sequence mutations that result in diarrhoea due to an intestinal endocrinopathy in patients and a validated mouse model [4].

### 2.4. Defects in Intestinal Immune Homeostasis

Monogenic inflammatory bowel disorders comprise a heterogeneous group of pathologies presenting as neonatal, infantile or very early-onset inflammatory bowel disease. The reader is referred elsewhere for more comprehensive reviews [7,39]. In essence, these disorders can be classified into (i) epithelial barrier and response defects, (ii) neutrophil granule dysfunction, (iii) hyperinflammatory and autoinflammatory disorders, (iv) defects in T- and B-cell function and (v) defects in regulatory T cells and IL-10 signalling [6]. IBD-like pathology in children aged under 2 years is a key pointer towards a monogenic disorder. The current diagnostic approach, after precise delineation of phenotype including family background, disease distribution (within and without the gastrointestinal tract), histological features and, if indicated, radiological features, is usually to undertake some limited functional screening followed by a genetic confirmation strategy. Increasingly, whole-exome and whole-genome screening are being used, and it seems very likely that genetic screening will be the primary investigation in the future. Immunological functional screening should include full blood count; neutrophil oxidative burst assay; immunoglobulins G, A, M, and E; lymphocyte subsets; FOXP3+CD25+CD24+ T cells; and XIAP (X-linked inhibitor of apoptosis protein). Anti-enterocyte antibodies are an important part of this screen if a reliable assay is available locally.

(a) Epithelial barrier and response defects

(i) TTC7A deficiency

TTC7A is a scaffolding protein expressed in intestinal epithelial cells and lymphocytes that binds a phosphatidylinositol kinase to the plasma membrane. TTC7A deficiency is heterogeneous depending upon the nature of the gene mutation. Phenotypic features can include intestinal epithelial apoptosis with mucosal inflammation, multiple intestinal atresias and combined immunodeficiency [40]. Untreated severe phenotypes carry a very high mortality in the first year of life. TTC7A appears to be an important regulator of epithelial polarity and adhesion, and so in TTC7A deficiency, intestinal biopsies will often demonstrate increased epithelial apoptosis and epithelial cell lifting off the basal membrane, resulting in the loss of barrier function and triggering of a mucosal inflammatory response.

TTC7A-deficient patients can have (progressive) multiple intestinal atresia (MIA) affecting any part of the intestine from the pylorus to the rectum. Approximately 50% of TTC7A-deficient patients have evidence of a combined immunodeficiency, and patients requiring PN support because of MIA have frequent central line sepsis. Up to 10% of TTC7A-deficient patients have VEOIBD without evidence of MIA or CID. This group frequently has early-onset secretory diarrhoea with or without haematochezia. Patients surviving beyond the first few years of life appear to have more extraintestinal manifestations, including epidermal hyperplasia and ichthyosis. 

Treatment of the severe phenotypes of this disorder is challenging, as the inflammatory bowel disorder is typically refractory to immunosuppressive therapies, including steroids and biologics. Those with CID may be candidates for regular immunoglobulin infusions and HSCT in due course. Where there has been progressive loss of intestinal luminal integrity because of progressive structuring, it might be necessary to consider bowel +/− liver transplantation after the HSCT. Recent data suggest that leflunomide is a potential disease-modifying agent in TTC7A deficiency, and clinical trials are pending [41].

(b) Neutrophil granule dysfunction

For the purpose of completion of this review, the dysfunction of neutrophil granules is mentioned here, but the reader is referred elsewhere for a discussion of chronic granulomatous disease(s).

(c) Autoinflammatory and hyperinflammatory disorders

Autoinflammatory diseases constitute a family of disorders defined by aberrant stimulation of inflammatory pathways without involving antigen-directed autoimmunity. Monogenic autoinflammatory syndromes include familial Mediterranean fever, tumour necrosis factor receptor-associated periodic fever syndrome (TRAPS), mevalonate kinase deficiency, cryopyrin-associated periodic fever syndromes (CAPS) and pyogenic arthritis pyoderma gangrenosum and acne (PAPA) syndrome.

(i) Mevalonate kinase deficiency (MKD)

MKD is usually associated with hyperimmunoglobulinaemia D and is characterised by febrile attacks, often with transient abdominal pain, diarrhoea, or vomiting. Case reports describe 2 patients presenting with severe neonatal colitis characterised by deep ulcerations and a dense neutrophilic infiltrate and were initially PN-dependent [42]. Both patients responded well to anti-IL-1 therapy with Anakinra.

(ii) XLP2/XIAP

X-linked lymphoproliferative syndrome 2 (XLP2) is caused by defects in the XIAP (X-linked inhibitor of apoptosis protein) gene. About a quarter of patients with XIAP deficiency manifest with a Crohn’s-like disease with a perianal fistulising phenotype that is largely resistant to conventional treatments. Recent studies have highlighted a frequent occurrence of XIAP variants in adult males with paediatric-onset CD.

XIAP deficiency is mainly associated with familial hemophagocytic lymphohistiocytosis (HLH), for which allogeneic hematopoietic stem cell transplantation (HSCT) is currently the only successful treatment strategy. A recent case report has described a child presenting with VEOIBD aged 3 weeks who was resistant to conventional treatments and had a whole-gene deletion of XIAP [43].

(iii) Familial haemophagocytic lymphohistiocytosis type 5 (FHL-5)

Familial haemophagocytic lymphohistiocytosis type 5 (FHL-5) is a genetically determined hyperinflammatory syndrome caused by an uncontrolled immune response mediated by T-lymphocytes, natural killer (NK) cells and macrophages. Type 5 FHL is due to mutations in the gene encoding syntaxin binding protein 2 (STXBP2). The most common presenting features are fevers, pronounced hepatosplenomegaly and cytopenias. Biochemical features include hyperferritinaemia, hypertriglyceridaemia and hypofibrinogenaemia, together with raised blood-soluble IL-2R (CD25). Early-onset disease can be associated with severe diarrhoea [44].

(d) Defects in T- and B-cell function

There are many causes of perturbed B-cell or T-cell activation that can result in immunodeficiency, autoimmunity or intestinal inflammation. These include common variable immunodeficiency [45], agammaglobulinaemia [46], hyper IgM syndrome [47], Wiskott–Aldrich syndrome [48] and SCID variants (see below). The reader is referred elsewhere for a full discussion. Other T-cell immunodeficiencies associated with IBD include:

(i) RIPK1 deficiency

Receptor-interacting serine/threonine-protein kinase 1 (RIPK1) is a critical regulator of cell death and inflammation. Patients with biallelic RIPK1 deficiency present with life-threatening combined immunodeficiency and/or intestinal inflammation associated with impaired lymphocyte functions, increased inflammasome activity and altered TNFα-mediated epithelial cell death responses [49]. Histologic features of colonic biopsies from affected patients with VEOIBD include pancolitis with ulcers and granulomata, chronic-active inflammation with mucosal erosions and epithelial degeneration.

(e) Defects in regulatory T cells and IL-10 signalling

(i) X-linked immune dysregulation, polyendocrinology enteropathy syndrome (IPEX)

IPEX is a rare monogenic primary immunodeficiency caused by mutations in the gene FOXP3, which is an essential transcription factor for thymic-derived regulatory T-cell function. Loss-of-function FOXP3 mutations result in a failure to suppress effector T cells, resulting in autoimmunity and enteropathy with colitis, as well as autoantibodies against enterocytes and/or goblet cells. Antibodies against harmonin (anti-AIE75 kDa), an enterocyte brush border scaffolding protein, appear to be specific to IPEX.

The classic presentation of IPEX is with multiorgan autoimmunity, including a severe enteropathy thought to have started in utero, type 1 diabetes and dermatitis. A recent review of the experiences of 30 families with IPEX seen over a 35-year period in France has reported that 100% of patients experience diarrhoea during the course of their disease, and in 68%, this was the first symptom [50]. In other patients, type 1 diabetes was the first symptom at a median age of 1.5 months. Additionally, 35% developed severe food allergy with elevated food-specific IgE in the blood, 91% of patients had a markedly elevated IgE (up to 12,000 kUI/L, normal <40) and 75% had eczematous lesions of the skin. Autoimmune thyroid disease and nephritis were also seen.

The authors described IPEX as phenotypically very heterogeneous, although they noted that mutations in the first intron of FOXP3 could produce atypical attenuated phenotypes and that mutations in the forkhead domain were associated with a poor prognosis.

HSCT is the current treatment of choice.

(ii) IL-2 receptor a chain defects (IL2RA encoding CD25)

CD25 is essential for T_REG_ function and also for the immune response, including the response to infective agents. Individuals with loss-of-function mutations have an IPEX-like picture and an impaired ability to fight infections.

(iii) LRBA and CTLA4 deficiency

LPS-responsive beige-like anchor protein (LRBA) and cytotoxic T lymphocyte-associated antigen 4 (CTLA4) give rise to broadly similar phenotypes due to their functional interactions. LRBA deficiency is associated with T_REG_ depletion and impaired T_REG_ cell-mediated suppression because of a profound deficiency of CTLA4. Patients present with hypogammaglobulinaemia, recurrent infections, enteropathy and multiple polyautoimmune manifestations, including type 1 diabetes, autoimmune haemolytic anaemia, autoimmune thyroiditis and polyarthritis [51].

(iv) IL-10 and IL10R deficiency

Loss-of-function defects in IL10 or its receptors (IL10RA and IL10RB) result in very early-onset inflammatory bowel disease presenting in the first months of life with an enterocolitis, folliculitis and perianal disease, including fissures, fistulae and abscesses (image). The phenotype is a result of a lack of functional anti-inflammatory effects of the IL10 signalling pathway. Defects in IL10 signalling predispose to B-cell lymphoma. In one series, 70% presented within the neonatal period and 95% within the first 6 months; 94% of infants had perianal lesions, 34% oral ulceration and 52% skin rash [52]. Children with IL10RB mutations had a much higher rate of B-cell lymphoma (32%).

Conventional anti-inflammatory treatments, including systemic steroids and monoclonals, are usually ineffective; hence, the definitive treatment for the disease is allogeneic HSCT.

A summary of the CODE discussed is shown in Table 1.

## 3. Conclusions

Infants with CODE remain clinically challenging. The early onset of severe diarrhoea can, if left untreated, lead to life-threatening dehydration. Advances in the understanding of the underlying pathogenesis and whole-exome and -genome sequencing have resulted in a number of new diagnoses, many being monogenetic. Common causes of diarrhoea in the newborn period and infancy should be ruled out first, followed by a structured diagnostic approach to allow for an early diagnosis and subsequent management. Many of the CODE subtypes result in the need for prolonged or indefinite parenteral nutrition. Further research is needed to develop new targeted therapies and potentially a long-term cure. There is a call for international collaboration and a registry to obtain prospective data, allowing a better understanding of long-term outcomes.

## Figures and Tables

**Table 1:** Summary of CODE.

Defects in Digestion and Absorption of Nutrients or of Ionic Transport	Defects in Enterocyte Structure and Function	Defects in Enteroendocrine Cell Differentiation	Defects in Intestinal Immune Homeostasis
**Congenital chloride-losing diarrhoea** **Congenital sodium diarrhoea** **Glucose–galactose malabsorption** **Congenital lactase deficiency** **Sucrose-isomaltase deficiency** **DGAT1 deficiency** **Other defects in intestinal epithelial lipid re-esterification and export** ➢ Abetalipoproteinaemia ➢ Hypobetalipoproteinaemia ➢ Chylomicron retention disease	**Microvillus inclusion disease** **Tufting enteropathy** **Trichohepatoenteric syndrome** **Syndromic protein-losing enteropathies with early-onset diarrhoea** ➢ Plasmalemma vesicle-associated protein mutations ➢ Hennekam lymphangiectasia–lymphoedema syndrome ➢ Congenital disorder of glycosylation tybe 1b ➢ CHAPLE syndrome ➢ Infantile systemic hyalinosis	**Enteric anendocrinosis** **Mitchel Riley syndrome** **ARX deficiency** **Proprotein convertase 1/3 deficiency (enteric dysendocrinosis)** **ICR-related gastrointestinal endocrinopathy**	**Epithelial barrier and response defects** ➢ TTC7A deficiency **Neutrophil granulocyte dysfunction** ➢ Chronic granulomatous disease(s) **Autoinflammatory and hyperinflammatory disorders** ➢ Mevalonate kinase deficiency ➢ XLP2/XIAP ➢ Familial haemophagocytic lymphohistiocytosis type 5 **Defects in B- and T-cell function** ➢ RIPK1 deficiency **Defects in regulatory T cells and IL-10 signalling** ➢ IPEX ➢ IL-2 receptor chain defects (IL2RA encoding CD25) ➢ LRBA and CTLA4 deficiency ➢ ILF-10 and IL10R deficiency

## Data Availability

A literature search was performed from 1992 to 2022 using Pubmed, MEDLINE and Cochrane Database of Systematic Reviews and recent guidelines reviewed. In the absence of evidence, recommendations reflect the authors’ expert opinion.
